# The Intron Retention Variant *CsClpP3m* Is Involved in Leaf Chlorosis in Some Tea Cultivars

**DOI:** 10.3389/fpls.2021.804428

**Published:** 2022-01-28

**Authors:** Xueyin Luo, Mengxian Zhang, Pei Xu, Guofeng Liu, Shu Wei

**Affiliations:** ^1^State Key Laboratory of Tea Plant Biology and Utilization, Anhui Agricultural University, Hefei, China; ^2^Henan Provincial Key Laboratory of Tea Plant Biology, Xinyang Normal University, Xinyang, China

**Keywords:** *Camellia sinensis*, albino tea, intron retention, ClpP3, dose-dependent effect

## Abstract

Tea products made from chlorotic or albino leaves are very popular for their unique flavor. Probing into the molecular mechanisms underlying the chlorotic leaf phenotype is required to better understand the formation of these tea cultivars and aid in future practical breeding. In this study, transcriptional alterations of multiple subunit genes of the caseinolytic protease complex (Clp) in the chlorotic tea cultivar ‘Yu-Jin-Xiang’ (YJX) were found. Cultivar YJX possessed the intron retention variant of *ClpP3*, named as *CsClpP3m*, in addition to the non-mutated *ClpP3*. The mutated variant results in a truncated protein containing only 166 amino acid residues and lacks the catalytic triad S182-H206-D255. Quantitative analysis of two CsClpP3 variants in different leaves with varying degrees of chlorosis in YJX and analyses of different chlorotic tea cultivars revealed that the transcript ratios of *CsClpP3m* over *CsClpP3* were negatively correlated with leaf chlorophyll contents. The chlorotic young leaf phenotype was also generated in the transgenic tobacco by suppressing *ClpP3* using the RNAi method; complementation with non-mutated *CsClpP3* rescued the wild-type phenotype, whereas *CsClpP3m* failed to complement. Taken together, CsClpP3m is involved in leaf chlorosis in YJX and some other tea cultivars in a dose-dependent manner, likely resulting from the failure of Clp complex assembly due to the truncated sequence of CsClpP3m. Our data shed light on the mechanisms controlling leaf chlorosis in tea plants.

## Introduction

Leaf pigments such as chlorophyll, carotenoids, and flavonoids (anthocyanins) are leaf color determinants. Changes in their abundances may lead to distinct leaf colors (Palmer and Mascia, 1980; Jiang et al., 2013; Zhao et al., 2017). In addition, chlorophyll and carotenoids present in chloroplasts function as photosynthetic substances, receiving light energy for carbon assimilation. Changes in their abundances can lead to dramatic changes in leaf photosynthetic capacity and efficiency, plant metabolism, responses to environmental fluctuation, growth, and development.

The majority of about a 100 tea cultivars/genotypes reported so far in China (Wang et al., 2015) exhibit chlorotic or albino young leaves, which is most likely due to a significant reduction in abundances of leaf chlorophylls, and also probably in carotenoids (Feng et al., 2014; Liu et al., 2017). The phenotypic development of these chlorotic leaves and initiation of their regreening are largely dependent on environmental conditions such as light intensity and temperature (Du et al., 2009; Wang et al., 2015; Shin et al., 2018). These tea cultivars are very different from some other plant mutants such as variegated barley (Li et al., 2019) and *golden leaf* cucumber (Gao et al., 2016), in which leaves possess abnormal color but do not regreen. As reported previously in this lab, leaves of *Camellia sinensis* var. *sinensis* cultivar ‘Yu-Jin-Xiang’ (YJX) are pale or yellow at an early developmental stage but turn green at the mature stage or later in the growing season (Liu et al., 2017). Leaf color conversion from a young pale/yellow leaf to a mature green leaf can be induced by shading treatment (Liu et al., 2017). Cultivars with convertible leaf color are highly desired by the tea industry because mature green leaves can maintain plant growth and regular productivity in successive years, while young pale (virescent) tea leaves can be used to produce a high-quality green tea with an enhanced savory (“umami”) taste and reduced astringency (Feng et al., 2014).

Extensive multi-omics studies have recently been conducted and reviewed along with many other findings related to these tea cultivars with abnormal leaf colors (Zhang et al., 2020). It has been well documented that young chlorotic or albino leaves usually contain significantly reduced chlorophylls and carotenoids (Liu et al., 2017), but contain enhanced levels of the non-protein amino acid theanine and reduced levels of catechins (Du et al., 2009; Wei et al., 2012; Feng et al., 2014; Liu et al., 2017). Catechins and their derivatives contribute to the bitter/astringent taste (Chaturvedula and Prakash, 2011), while theanine contributes to the umami taste and counteracts the astringent and bitter taste in tea infusions (Ashihara, 2015). Thus, teas made from such chlorotic young leaves have reduced astringency and enhanced umami flavor. Moreover, mega-data accumulated from omics analyses indicated that numerous pathways at transcriptomic and proteomic levels have been affected in these chlorotic tea cultivars, including data related to chlorophyll and carotenoids, phenylpropanoid/flavonoid metabolism, high light stress-induced responses, signal transduction of reactive oxygen species, imbalanced nitrogen and carbon, and posttranslational modification (Gao et al., 2016). However, genetic mutations directly involved in, or responsible for leaf chlorosis in these cultivars have yet to be elucidated.

Leaf color mutation largely results from defective genes related to chlorophyll and carotenoid biosynthesis, chloroplast biogenesis, and activities (Pogson and Albrecht, 2011). Protein mutation experiments indicate that numerous nuclear-encoded chloroplast proteins are able to affect chloroplast biogenesis and leaf color in *Arabidopsis* (Myouga et al., 2013) and maize (Belcher et al., 2015). The majority of these defective genes result in lethal consequences, and some lead to aberrant chloroplast development and irreversible leaf color change (Myouga et al., 2013). However, a group of mutants in *Arabidopsis* (Kim et al., 2009), tobacco (Moreno et al., 2017), rice (Dong et al., 2013), and maize (Xing et al., 2014) have been reported to possess a similar leaf color phenotype as found in chlorotic tea cultivars, exhibiting green-revertible chlorotic leaf color. All these mutants share a defective subunit of the complex of plastidial caseinolytic protease (Clp). The Clp proteolytic system can remove misfolded and damaged proteins in plastids (Olinares et al., 2011b). It plays a vital role in chloroplast biogenesis and development (Nishimura and Van Wijk, 2015) and is essential for chloroplast proteostasis through regulating the degradation of substrate proteins, including enzymes involved in the production of tetrapyrroles and isoprenoids such as chlorophylls and carotenoids (Rodriguez-Concepcion et al., 2019). The Clp protease complex consists of a heptameric P-ring as its catalytic core, an R-ring, and other component proteins (Kress et al., 2009). Malfunction of any of four members of the P-ring, ClpP3, ClpP4, ClpP5, and ClpP6, each with proteolytic activity, may be lethal or lead to virescent leaves (Kim et al., 2013). Moreover, it has been noted that suppression of some of the Clp protease complex genes can result in transcriptional alterations of multiple other Clp subunit genes (Kim et al., 2009; Dong et al., 2013; Moreno et al., 2017). In our previous study, we found that the expression of *ClpP5* is remarkably enhanced in the chlorotic tea cultivar YJX compared with the regular green leaf control cultivar (Liu et al., 2017). These findings suggest that a dysfunctional Clp complex could be responsible for chlorotic leaf phenotype in YJX.

In this study, transcriptional levels and sequencing data of all P-ring genes in the chlorotic tea cultivar YJX and the green tea cultivar ‘Shu-Cha-Zao’ (SCZ) were compared in order to uncover defective P-ring genes in YJX. Our data indicated that an intron retention variant of *CsClpP3* and *CsClpP3m* resulted in a mutated and malfunctional ClpP3 protein. Its transcript levels were related to the degree of leaf chlorosis in five tested chlorotic tea cultivars in a dose-dependent manner. Transgenic studies revealed that *ClpP3* suppression led to the chlorotic leaf phenotype in transgenic tobaccos, which were complemented by overexpressing wild-type (WT) tea *ClpP3*, but not by overexpressing *ClpP3m*. Our findings have set a basis for further investigation on the mechanisms controlling leaf chlorosis in tea plants and practical breeding for elite chlorotic tea cultivars.

## Materials and Methods

### Plant Materials

**FIGURE 1:**
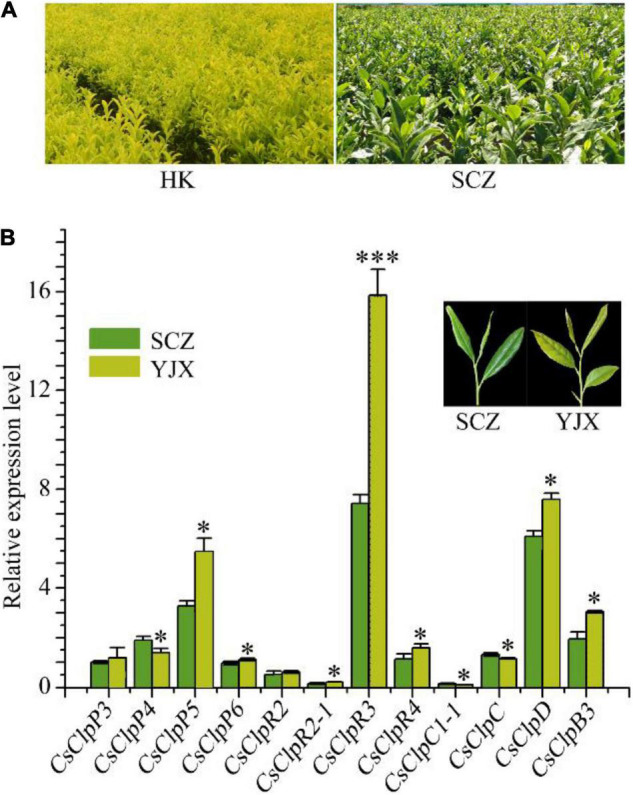
Chlorotic tea garden and transcriptional alteration of the Clp complex genes in the chlorotic leaves of tea cultivar YJX compared with normal cultivar SCZ. **(A)** chlorotic tea garden of cv. ‘Huang-Kui’ (HK) compared with regular green leaf cv. ‘Shu-Cha-Zao.’ **(B)** Transcriptional alteration of the Clp complex genes in the chlorotic leaves of tea cultivar YJX compared with normal cultivar SCZ. YJX, ‘Yu-Jin-Xiang’; SCZ, ‘Shu-Cha-Zao.’ Statistical analysis using Student’s *t*-test was performed in at least triplicate using SPSS19 software. *p < 0.05; ***p < 0.001.

**FIGURE 2:**
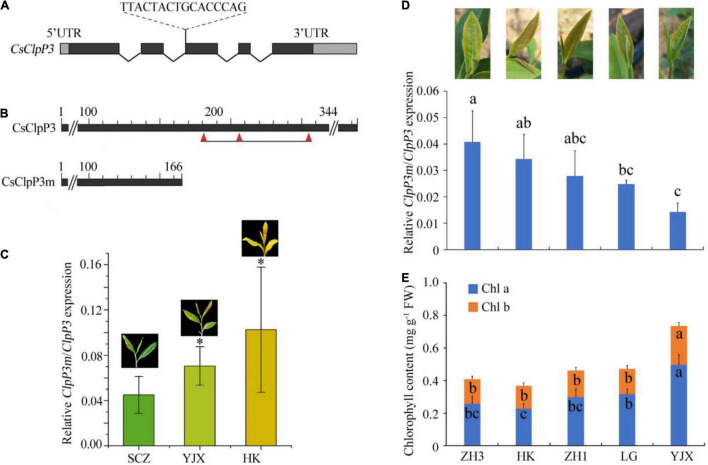
Gene structure of CsClpP3 and its intron retention variant CsClpP3m and their transcript levels in tea leaves. **(A)** The intron retention site and fragment of *CsClpP3*. **(B)** Prediction of active sites for deduced ClpP3 and ClpP3m. Three red triangles refer to the Ser-His-Asp residues that form the ClpP3 catalytic triad. **(C)** Relative transcript levels of *ClpP3m* to these of *ClpP3* in three tea cultivars with different chlorotic leaf phenotypes in early spring. Red squares on the leaves mark the chosen colors for the corresponding columns. **(D)** Transcript level ratio of *ClpP3m* to *ClpP3* in cultivars ZH3, HK, ZH1, LG, and YJX in summer. **(E)** Chlorophyll contents in cultivars ZH3, HK, ZH1, LG, and YJX. SCZ, ‘Shu-Cha-Zao’; YJX, ‘Yu-Jin-Xiang’; HK, ‘Huang-Kui’; ZH1, ‘Zhong-Huang1’; ZH3, ‘Zhong-Huang3’; LG, ‘Le-Guan.’ One way-ANOVA analysis was performed using SPSS19 software. “*” or different letters denote *p* < 0.05.

Tea plants of *C. sinensis* var. *sinensis* cv. chlorotic cultivars ‘Yu-Jin-Xiang’ (YJX), ‘Huang-Kui’ (HK), ‘Zhong-Huang1’ (ZH1), ‘Zhong-Huang3’ (ZH3), ‘Le-Guan’ (LG), and normal green cultivar ‘Shu-Cha-Zao’ (SCZ) were grown at the Experimental Tea Garden of the Anhui Agricultural University in Da-Yang-Dian, Hefei, China. Tender shoots with two unfolded leaves of chlorotic and normal color cultivars (Figures 1, 2C,D) were excised in early spring and summer (August 17, 2020) for use with analyses of ClpPR genes and chlorophyll abundances. *Nicotiana tabacum* cv. ‘Yun Yan 85’ plants were grown in pots containing a peat/vermiculite mixture (1/3, v/v) in a growth chamber under a 16-h photoperiod with a 20/25°C night/day temperature and were regularly fertilized using Peters Professional 20–20–20 GP (ICL Specialty Fertilizers, Summerville, SC, United States).

### RNA and DNA Extraction

Total RNA was extracted from leaf samples of tea and tobacco plants using Takara RNAzol reagent, and genomic DNA contamination was removed with DNaseI (Takara, Dalian, China). RNA quality and quantity were determined using both agarose gels and a NanoDrop 2000 spectrophotometer (Thermo Fisher Scientific, Wilmington, DE, United States). RNA samples with *A*_260/230_ ratio between 2.0 and 2.2 were used for further studies. Plant genomic DNA was isolated using an Easy Pure Plant Genomic DNA Kit (TransGen, Beijing, China) according to the instructions of the manufacturer.

### Gene Cloning and Sequence Analysis

Sequence information of tea Clp complex subunits used in this study was obtained from our previous transcriptomic data (Liu et al., 2017) and tea genomic sequence information (Xia et al., 2019). Sequences of tobacco *NtClpP3a* and *NtClpP3b* were obtained according to the previously published literature (Moreno et al., 2017). Full-length cDNA was cloned with specific primers (Supplementary Table 1) and inserted into pEASY-T1 (TransGen, Beijing, China) for sequencing. COBALT protein sequence alignment analyses were conducted using the BLATA tool at the National Center for Biotechnology Information.^1^ Intron retention of *CsClpP3* was found in one of the two sequenced samples and confirmed with re-sequencing data. Active site prediction of the deduced CsClpP3 and CsClpP3m proteins was conducted using Hmmer (version 3.2.1).^2^

### Construction of *NtClpP3* Knock-Down Tobacco Plants

Since tobacco contains two *ClpP3* homologs, *ClpP3a* and *ClpP3b*, the alignment of the two coding sequences was conducted. A highly similar fragment (443 bp) starting from 453 to 896 bp of *NtClpP3a* with only three mismatched based at 603, 669, and 678 between *NtClpP3a* and *NtClpP3b* was PCR cloned as the sense and antisense fragments for an RNAi construct to knock down both homologs. A recombinant cloning approach was applied using the intermediate vector pHANNIBAL (Wesley et al., 2001). The sense and antisense fragments of *NtClpP3* were PCR retrieved using primers designed with the free program CE Design (version 1.04) (Vazyme, Nanjing, China) and were, respectively, inserted at the *Xho*I/*Kpn*I and *Bam*HI/*Hin*dIII sites of pHANNIBAL. Then, the RNAi expression cassette was isolated from pHANNIBAL using *Not*I and ligated into the *Not*I site of the binary vector pART27 (Gleave, 1992). The resultant vector was then transformed into *Agrobacterium tumefaciens* (GV3101). Standard *Agrobacterium*-mediated tobacco leaf disk transformation was conducted to generate *NtClpP3* knock-down plants. Transgenic and non-transgenic plants grown under the same conditions were then evaluated for growth, phenotype, and transcription of Clp subunits and used for complementation assays as detailed below.

### Complementation Assay of *NtClpP3* Knock-Down Plants Using *CsClpP3* and *CsClpP3m*

Coding sequences of *CsClpP3* and mutant *CsClpP3m* were, respectively, isolated from the normal green cultivar SCZ and the chlorotic cultivar YJX and were separately subcloned into the *Xba*I/*Sac*I sites downstream of the Cauliflower mosaic virus 35S promoter of binary vector pPZP121 (Hajdukiewicz et al., 1994) with recombinant cloning. The constructs pPZP121-CsClpP3 and pPZP121-CsClpP3m were confirmed by DNA sequencing, and then the confirmed binary vectors were separately introduced into *A. tumefaciens* strain GV3101 for generation of transgenic tobacco.

### Construction of the Transgenic Tobaccos Overexpressing *CsClpP3* and *CsClpP3m*

GV3101 colonies containing constructs pPZP121-CsClpP3 and pPZP121-CsClpP3m were also used to generate *CsClpP3* and *CsClpP3m* overexpression plants through *Agrobacterium*-mediated leaf disk transformation as mentioned earlier. Transgenic plants grown under the same conditions were then evaluated for growth, phenotype, and transcription of Clp subunits.

### Observation of Chloroplast Ultrastructure and Measurements of Chlorophyll Contents

Observation of chloroplast ultrastructure and measurements of chlorophyll contents were carried out according to our previous study (Liu et al., 2017). In brief, fresh leaves were excised and infiltrated with 4% glutaraldehyde solution using a syringe. Infiltrated leaves were cut into 2 mm × 2 mm pieces and were further sectioned using a TCS CM1900 freezing microtome (Leica, Germany). The ultrathin sections were double lead stained according to a previously published protocol (Daddow, 1983) and were then observed using an HT-7700 transmission electron microscope (TEM) (Hitachi, Japan).

Chlorophyll was extracted overnight using a 10-ml extraction solution (5% acetone: 95% ethanol, v/v) until the sample leaves became completely white. The extract was then measured using a UV spectrophotometer (U-5100, Hitachi, Japan) at A645 and A663. Three biological and technical replicates were employed. The chlorophyll contents were calculated as in our previous study (Liu et al., 2017).

### Quantitative Real-Time PCR

To examine transcript levels of the Clp protease system genes, gene-specific primers were designed for 10 subunits of the P- and R-rings of the Clp complex based on our own transcriptomic data (Liu et al., 2017) and genome information (Xia et al., 2019), except for the plastidial gene *ClpP1* (Kuroda and Maliga, 2003). For transcript quantification of tea CsClpP3 and its variant, quantitative real-time PCR (qPCR) assays were performed using two pairs of primers. The primer qPCR-CsClpP3m-F (Supplementary Table 1) containing the intron retention fragment was used for *CsClpP3m* transcript level evaluation. The pair of primers targeted the common sequences present in both *CsClpP3* isoforms (qPCR-CsClpP3m-F and -R; Supplementary Table 1) but absent from *NtClpP3a* and *NtClpP3b* and was used to quantify their transcript levels collectively. The transcript level of CsClpP3 was obtained by calculating the difference between the levels with the two pairs of primers. To quantify transcript levels of *NtClpP3*, sequences present in both *NtClpP3a* and *NtClpP3b* but absent from *CsClpP3m* were used for primer design (Supplementary Table 1). For data normalization, 18*S* rRNA was used as the reference gene. qPCR assays were conducted on a CFX96 platform (Bio-Rad) using gene-specific primers (Supplementary Table 1) and Top Green qPCR SuperMix (TransGen, Beijing, China) according to the instructions of the manufacturer. Transcript levels were calculated using the 2^–ΔΔCT^ method. Three biological and technical replicates were performed for each experiment.

### Statistical Analysis

In this study, all data were obtained from at least three biological and technical replicates. One-way ANOVA analysis and *t*-test were performed using SPSS19 software.^3^

## Results

### Expression Analysis of Caseinolytic Protease Subunit Genes in Control and Albino Tea Leaves

Chlorotic tea cultivars are now widely used in the tea industry on a large scale (Figure 1A); thus, it is of greater urgency to uncover the mechanisms of tea leaf chlorosis. In our previous study, a dramatically high transcript level of *CsClpP5* was found in virescent leaves of YJX relative to that of the green leaf cultivar SCZ (Liu et al., 2017), suggesting an abnormal transcriptional alteration in YJX leaves. In this study, the expression of all the genes in the P- and R-rings was further examined using qRT-PCR. Our results confirmed that the transcript level of *CsClpP5* in virescent leaves of YJX was significantly higher than that of SCZ (Figure 1B). In addition, compared with normal green tea leaves of SCZ, YJX had significantly higher expression levels of *CsClpP6*, *CsClpR2-1*, *CsClpR3*, *CsClpR4*, *CsClpD*, and *CsClpB3* (*p* < 0.05) and significantly lower levels of *CsClpP4* and *CsClpC1* (Figure 1B). However, transcript levels of *CsClpP3* and *CsClpR2* in the two cultivars were not significantly affected (Figure 1B). These results indicated that transcriptional alterations occurred to the majority of the Clp subunits in genes of YJX through compensation with other nuclear encoding plastidial chaperones, especially *CsClpB3*, suggesting that the Clp complex in YJX was likely defective and non-functional.

### Identification of the Intron Retention Isoform of *CsClpP3* and *CsClpP3m*

Since the Clp complex in YJX could be malfunctioning and subunit protein stoichiometry of the Clp complex is thought to be critical for its assembly and function (Olinares et al., 2011a), catalytic P-ring subunit genes were first cloned in this study to check for differences in cDNA and protein sequences between chlorotic YJX and normal SCZ. From the recently released tea genome information at the Tea Plant Information Archive (TPIA)^4^ (Xia et al., 2019), single copies of *ClpP3* (TEA013179) and *ClpP5* (TEA023304) and two copies of *ClpP4* (TEA024098) and *ClpP6* (TEA022442) were found in tea genome after manual annotation confirmation using the database at the National Center for Biotechnology Information (NCBI) (see text footnote 1). The P-ring subunits were first cloned from both SCZ and YJX and then sequenced. The two cDNA copies of *ClpP4* in tea plants, named *ClpP4-1* (GenBank Acc. Nos. MT940097 and MT940098 for SCZ and YJX, respectively) and *ClpP4-2* (MT940099 and MT940100 for SCZ and YJX, respectively), were isolated, and their protein sequences were deduced. For ClpP4-1 proteins, there were mismatches at positions 18 (Phenylalanine in SCZ and Tyrosine in YJX) and 283 (Phenylalanine in SCZ and Leucine in YJX) between YJX and SCZ. COBALT progressive multiple alignments (Papadopoulos and Agarwala, 2007) of 415 protein sequences from the NCBI dataset revealed that the position 18 mismatch is not within the highly conserved region, which starts from position 97. In many ClpP4 proteins from different plant species, the amino acid residue Leucine often appears at position 283. For ClpP4-2 and the single-copied ClpP5 in the tea genome (MT940101 and MT940102, respectively, for SCZ and YJX), no mismatches were detected in either protein or cDNA sequences between YJX and SCZ. For ClpP6, one transcript TEA022442, annotated as Clp protease proteolytic subunit 6 isoform 1 (*ClpP6-1*) at the TPIA, was found in both SCZ (MT940103) and YJX (MT940104). No differences in protein sequences of ClpP6-1 were found between SCZ and YJX. However, cloning of the transcript of Clp protease proteolytic subunit 6 isoform 2 (*ClpP6-2*) (XM_028264497.1) failed, as the fragments from both SCZ and YJX were lacking 93 bp normally present at 673–765 bp of *ClpP6-1*. These data indicated that those cloned P-ring genes were unlikely responsible for the malfunctional Clp complex because the protein sequences from chlorotic and green tea cultivars SCZ and YJX were identical or similar.

A single copy of the *ClpP3* gene was found in YJX (MT944096) and SCZ (MT944095). Three differences in their amino acid sequences were found between CsClpP3-YJX and CsClpP3-SCZ, which were positions 16 (histidine in SCZ and arginine in YJX), 48 (aspartic acid in SCZ and glutamic acid in YJX), and 342 (alanine in SCZ and threonine in YJX). COBALT progressive multiple alignments (Papadopoulos and Agarwala, 2007) of 397 protein sequences from the NCBI dataset revealed that all three variant amino acid residues are not within the highly conserved region and differ considerably in many ClpP3 proteins from different plant species. Moreover, in the chlorotic tea cultivar YJX, an isoform of *CsClpP3* containing a 16 bp intron segment (TTACTACTGCACCCAG) was found in addition to the non-mutated WT *CsClpP3* gene and was named *CsClpP3m*. Further analysis showed that this intron retention occurred at the beginning of the third exon of *CsClpP3* (Figure 2A) and resulted in a premature termination codon, leading to a truncated ClpP3m with only 166 of the N-terminal amino acid residues, while non-mutated ClpP3 contained 344 amino acid residues (Figure 2B). Hmmer (version 3.2.1) (see text footnote 2) analysis revealed that CsClpP3m lost the serine–histidine–aspartic acid catalytic triad (S182-H206-D255), which comprises the serine-type proteolytic core of ClpP3 (Peltier et al., 2004). Interestingly, the same intron retention of *CsClpP3m* was also found in several other chlorotic cultivars ‘Huang-Kui’ (HK), ‘Zhong-Huang1’ (ZH1), ‘Zhong-Huang3’ (ZH3), and ‘Le-Guan’ (LG), whose young leaves were more yellowish than those of YJX.

### Analysis of *CsClpP3m*/*CsClpP3* With Tea Leaf Chlorotic Phenotype in Different Growing Seasons

To further investigate the involvement of *ClpP3m* in leaf chlorosis, the transcript levels of *CsClpP3m* in YJX and HK chlorotic leaves in early spring were determined using the qRT-PCR method with a pair of primers, one of which, CsClpP3m-466-F, contained the retained intron segment (Supplementary Table 1), and the normal green leaf cultivar SCZ was used as control. Interestingly, transcript levels of *CsClpP3m* relative to non-mutated *CsClpP3* in chlorotic leaves of YJX and HK were associated with the degree of chlorosis in tea leaves (Figure 2C). In the green leaf cultivar SCZ, transcripts of the mutated *CsClpP3m* were unexpectedly detected, but at a very low level. Moreover, transcript ratios of *CsClpP3m* over *CsClpP3* were significantly different (*p* < 0.05) in the summer growing season among the chlorotic tea cultivars HK, ZH1, ZH3, and LG, which all possessing different degrees of chlorosis but with the green-revertible leaf phenotype as observed in YJX (Figure 2D). Interestingly, the abundances of chlorophyll *a* and *b* were also different (*p* < 0.05) in the chlorotic leaves of these cultivars (Figure 2E). These results indicated that the transcript ratio of *CsClpP3m*/*CsClpP3* correlated negatively with the abundances of chlorophyll *a* (−0.901) and chlorophyll *a* + *b* (−0.887) and positively with the degree of leaf chlorosis as shown in Figure 2C. Our data indicated that the dose-effect of *CsClpP3m* on the chlorotic leaf phenotype existed in the tested cultivars YJX, HK, ZH1, ZH3, and LG.

### Functional Characterization of *CsClpP3* and *CsClpP3m* in Transgenic Tobacco

It is known that the ClpP3 null mutation leads to seedling lethality in *Arabidopsis* (Kim et al., 2013). Point mutation of the serine–histidine–aspartic acid triad of ClpP3 shows that the loss of its catalytic core does not result in any visible changes in plant growth and development (Liao et al., 2018). However, possible effects of truncated *CsClpP3* (*CsClpP3m*) lacking not only the catalytic core but also 178 amino acid residues, on leaf chlorosis needed to be further examined heterologously in tobacco plants since tea plants are difficult to genetically transform, which rules out endogenous gene expression through conventional *Agrobacterium*-mediated transformation approach or transient expression through infiltration. For this purpose, *CsClpP3* or *CsClpP3m* driven by cauliflower mosaic virus 35S promoter (35S) was introduced into tobacco plants. The transgenic plants had enhanced transcript levels of transgene *CsClpP3* or *CsClpP3m* as expected. Chlorophyll contents in the transgenic plants were also found to be significantly decreased (*p* < 0.05 or 0.01 or 0.001, Supplementary Figure 1). However, no visible difference in leaf color was noted between WT plants and transgenic plants overexpressing either *CsClpP3* or *CsClpP3m*.

**FIGURE 3:**
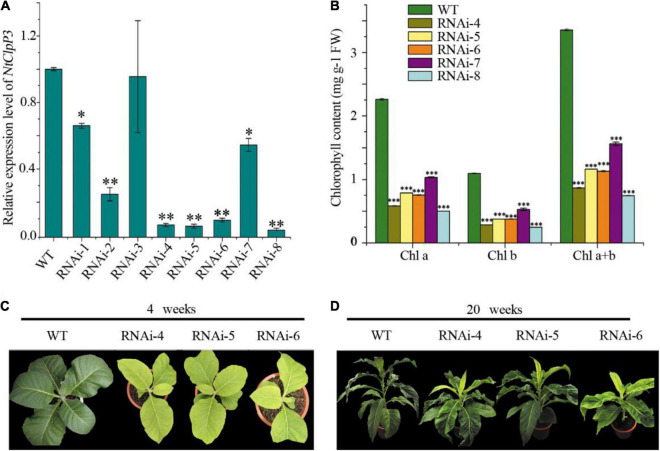
Transcriptional and morphological alterations of transgenic tobaccos with suppressed NtClpP3. **(A)** Transcript levels of *NtClpP3* in wild-type (WT) and transgenic tobaccos. **(B)** Chlorophyll contents in WT and transgenic tobaccos. **(C)** Differences in leaf appearances between 4-week-old WT and transgenic tobaccos. **(D)** Morphological differences between 20-week-old WT and transgenic tobaccos. One way-ANOVA analysis was performed using SPSS19 software. *p < 0.05; **p < 0.01; ***p < 0.001.

Overexpression of tea *CsClpP3* or *CsClpP3m* in WT tobacco failed to generate chlorotic leaf phenotype as observed in YJX likely due to the action of endogenous ClpP3 in tobacco. Therefore, transgenic tobaccos with collective suppression of two endogenous *ClpP3* genes (*NtClpP3a* and -*b* (Moreno et al., 2017) were generated using an RNAi-NtClpP3 expression cassette. Since the mRNA sequences of *NtClpP3a* and *NtClpP3b* in tobacco are highly similar (97%), a fragment (443 bp) from *NtClpP3a* used for the RNAi cassette construction was also identical to the corresponding section of *NtClpP3b*; thus, the resultant RNAi expression cassette (Supplementary Figure 2A) in the binary vector pART27 (Gleave, 1992) was expected to suppress both endogenous *NtClpP3a* and *NtClpP3b* in transgenic tobaccos. Strong and collective inhibition of *NtClpP3a* and *NtClpP3b* was found in different independent transgenic lines with some variations, compared with WT plants (*p* < 0.05 or 0.01) (Figure 3A). These lines had consistent and significant decreases in chlorophyll abundances (*p* < 0.001), which positively correlated with the corresponding transcript levels of *NtClpP3* genes (Figure 3B). The transgenic lines of RNAi–4, –5, and –6 all exhibited a strong inhibition of *NtClpP3* expression (Figure 3A), and the expected chlorotic leaf phenotype was observed in the leaves of 4-week-old transgenic plants carrying the RNAi-NtClpP3 expression cassette (Figures 3C,D). As the plants grew, the chlorotic leaves gradually turned green, but newly generated young leaves remained chlorotic (Figure 3D), which phenocopied the chlorotic leaf phenotype of YJX (Liu et al., 2017). Moreover, growth retardation and delayed flowering time were also observed in the transgenic tobaccos with suppressed *NtClpP3* in this study (Figure 3D). These lines were also employed for further analyses.

**FIGURE 4:**
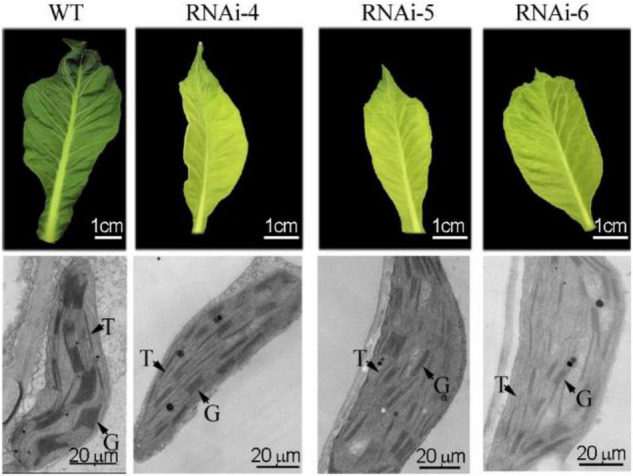
Ultrastructural observation of green and chlorotic leaves from non-transgenic WT and transgenic tobaccos with suppressed *NtClpP3*, respectively. T, thylakoid; G, grana.

Ultrastructures of the first unfolded leaves of WT and transgenic RNAi–4, –5, and –6 tobaccos were observed using a TEM. Results showed that the chloroplast structure in WT was well developed. The thylakoid membrane was obvious, and the grana were hypertrophic and compact (Figure 4). However, in chlorotic leaves of transgenic tobacco, the chloroplast structure was incomplete, the thylakoid membrane was not obvious, and the grana were flat and sparse (Figure 4).

**FIGURE 5:**
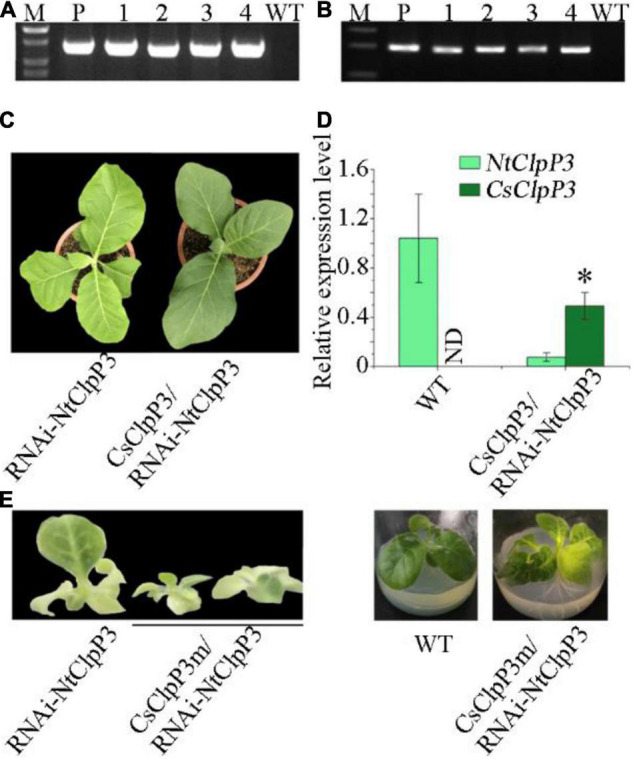
Complementation of transgenic tobaccos with RNAi-NtClpP3 using *CsClpP3* and *CsClpP3m* from chlorotic tea YJX. **(A)** PCR confirmation for the presence of the transgene cassette RNAi-NtClpP3. **(B)** PCR confirmation for the presence of the transgene *CsClpP3* from tea YJX. **(C)** Green leaf recovery of transgenic tobacco carrying RNAi-NtClpP3 complemented with tea *CsClpP3*. **(D)** Transcript levels of *NtClpP3* and *CsClpP3* in WT and complemented plants. **(E)** Leaf chlorosis phenotypes of 1-week-old and 6-week-old transgenic tobacco plants carrying RNAi-NtClpP3 complemented with tea *CsClpP3m*. ND, not detectable; one way-ANOVA analysis was performed using SPSS19 software. *p < 0.05.

Plant expression vectors pPZP121-CsClpP3 and pPZP121-CsClpP3m containing *CsClpP3-YJX* and *CsClpP3m*, respectively, all driven by 35S promoter, were constructed (Supplementary Figure 2B) and introduced into the transgenic plants carrying the RNAi-NtClpP3 cassette for genetic complementation through *Agrobacterium*-mediated transformation. Independent transgenic tobaccos containing stacked genes (RNAi-NtClpP3 and *CsClpP3* or *CsClpP3m*) were obtained. The presence of both transgene cassette RNAi-NtClpP3 (Figure 5A) and transgene *CsClpP3* (Figure 5B) were screened by PCR using gene-specific primers, and the PCR product was sequenced for confirmation. Complementation of the RNAi-NtClpP3 transgenic tobacco with tea *CsClpP3* gene resulted in the recovery of the green color from leaf chlorosis (Figure 5C). qRT-PCR analysis showed that in the RNAi-NtClpP3 transgenic plants complemented with tea *CsClpP3*, the transcript level of *CsClpP3* was high, while that of *NtClpP3* remained low (Figure 5D). However, the chlorotic leaf phenotype of the RNAi-NtClpP3 plant was not rescued by complementation with tea *CsClpP3m* (Figure 5E) as expected. No discernable differences in the young leaf chlorosis development and its old leaf regreening were found between the transgenic RNAi-NtClpP3 plants complemented with *CsClpP3m* and RNAi-NtClpP3 (Figures 3D, 5E). These results indicated that *CsClpP3* is essential for maintaining the normal green leaf phenotype in tobacco, whereas the truncated protein sequence produced by *CsClpP3m* was not able to rescue the chlorotic leaf phenotype. Taken together, our data indicated that *CsClpP3m* could result in the chlorotic leaf phenotype in tobacco.

**FIGURE 6:**
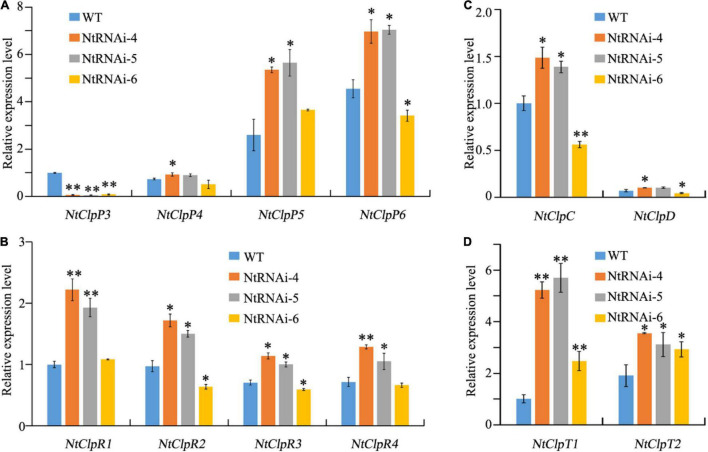
Transcriptional alterations of different Clp complex subunit genes in WT and transgenic tobaccos with suppressed *NtClpP3*. **(A)** Relative transcript levels of *ClpP3-6* P-ring genes in transgenics to that of *NtClpP3* in WT plants. **(B)** Relative transcript levels of R-ring *ClpR1-4* in transgenics to that of *NtClpR1* in WT plants. **(C)** Relative transcript levels of chaperone ring *ClpC* and -*D* in transgenics to that of *NtClpC* in WT plants. **(D)** Relative transcript levels of *ClpT1-2* in transgenics to that of *NtClpT1* in WT plants. One-way ANOVA and *t*-test analyses were performed to examine the significant differences in transcript levels between transgenic and WT tobaccos. *p < 0.05; **p < 0.01.

The expression levels of different Clp complex subunits in WT tobacco and transgenic *NtClpP3* knock-down lines were quantified using qRT-PCR to check whether transcriptional alterations of other Clp complex subunit genes were present in *NtClpP3* suppression lines, as we found in the chlorotic tea YJX. Our data indicated that general transcriptional levels of all subunits of the Clp complex were affected to different extents in the transgenic tobaccos with suppressed *NtClpP3* (Figure 6). Transcript levels of *NtClpP4*, –*5*, and –*6* from P-ring were largely enhanced in transgenic lines NtRNAi–4 and –5 (*p* < 0.05), but not in the line of NtRNAi-6, where the transcriptional levels of P-ring components were either suppressed or unchanged (Figure 6A). Similar transcriptional alterations were also noted for R-ring and chaperone members (Figures 6B,C), and all enhanced in the transgenic NtRNAi–4 and –5 (*p* < 0.05) but suppressed or unaffected in the line of NtRNAi-6. It was also noted that transcriptional levels of accessory protein members *NtClpT1* and *NtClpT2* were all enhanced (*p* < 0.05) in three transgenic lines at different extents (Figure 6D). Our results indicated that in the three transgenic lines with suppressed *NtClpP3*, transcript levels of *NtClpP5*, *NtClpT1*, and *NtClpT2* were significantly higher than those of the corresponding genes in WT tobacco, while the transcript levels of other Clp protease complex genes varied depending on transgenic lines. Our results indicated that a defective *ClpP3* gene results in transcriptional alterations of other Clp subunit genes in tobacco. Interestingly, transcript level variations of the different subunit genes were similar to those in the leaves of YJX, which strongly suggests a role of CsClpP3m in the formation of chlorosis of YJX.

## Discussion

Chlorotic tea cultivars such as YJX are widely grown for their tea products, which are highly appreciated for their reduced astringency and stronger umami flavor (Feng et al., 2014). Nevertheless, molecular mechanisms underlying their albino leaf color phenotype are not well understood, although extensive studies have been conducted (Gao et al., 2016). In this study, sequence analysis suggested that except for CsClpP3, all the P-ring protein sequences were either identical or highly similar between SCZ and YJX; thus, they are unlikely to be the cause of chlorotic leaves in YJX. Our data indicated that transcript levels of the variant *CsClpP3m* relative to those of non-mutated *CsClpP3* closely correlate with YJX leaf chlorosis and possibly also in another four chlorotic cultivars HK, ZH1, ZH3, and LG, which all exhibit different degrees of the chlorotic and green-revertible leaf phenotype. Such involvement has been demonstrated in a dose-dependent manner with different lines of evidence, which include the lack of a proteolytic triad in the truncated product of CsClpP3m, and the heterologous functional demonstration of CsClpP3m on leaf chlorosis in transgenic tobaccos.

The structure, assembly, and function of the Clp proteolytic system in plants have been gradually revealed (Adam et al., 2006; Nishimura and Van Wijk, 2015; Wei et al., 2015; Moreno et al., 2017; Welsch et al., 2018). The null mutation of *ClpP3* in *Arabidopsis* can lead to delayed embryo development and seedling lethality, and the mutant seedlings can survive and produce pale-green leaves only when exogenously supplemented with sucrose (Kim et al., 2013). Moreover, ClpP3 was found physically interacting with ClpP6 and ClpT in rice and plays a role in Clp complex assembly (Dong et al., 2013). Recently, it was found that the loss of ClpP3 proteolytic activity through point mutation of the catalytic core does not affect plant growth and development (Liao et al., 2018). These indicate that the observed phenotypic changes in a ClpP3 null mutant likely result from misassembly of the ClpP complex due to the absence of ClpP3 rather than the lack of ClpP3 proteolytic activity (Liao et al., 2018). However, it is unknown what defines the essential structure of ClpP3 required for Clp complex assembly. In this study, truncated CsClpP3m containing 166 amino acid residues at the N-terminus found in chlorotic YJX was unable to rescue pale-green leaf phenotype in the RNAi-mediated transgenic tobacco with suppressed *ClpP3*, indicating that CsClpP3m would not be able to interact with its Clp partners for the complex assembly and function. Further investigation will be conducted to determine the essential ClpP3 structure required for ClpP complex assembly.

Interestingly, transcript ratios of *CsClpP3m*/*CsClpP3* were positively correlated with the degrees of leaf chlorosis and negatively correlated with the corresponding chlorophyll abundances in chlorotic cultivars YJX, HK, ZH1, ZH3, and LG. Moreover, significantly higher expression of *CsClpB3* was noted in YJX chlorotic leaves compared with SCZ green leaf control, which was likely due to a compensatory effect from defective Clp protease activity, which has been elegantly demonstrated in *Arabidopsis* (Llamas et al., 2017). These data suggested a dose-dependent effect of *CsClpP3m* on tea leaf chlorosis. This hypothesis is firmly supported by previous studies on *AtClpP3* (Kim et al., 2013) and *AtClpP4* (Wei et al., 2015) in *Arabidopsis*, in which both genes showed clear dose-related effects on the degree of leaf chlorosis. Nevertheless, the dose-related effect of *CsClpP3m* on tea leaf chlorosis in YJX should be further investigated to clarify the quantitative threshold of the relative transcriptional levels of *CsClpP3m* over *CsClpP3* for leaf chlorosis development and initiation of regreening. Moreover, light intensity was shown to affect leaf chlorosis development (Liu et al., 2017). In this study, the RNAi tobacco lines exhibited chlorosis of young leaves only when placed at a high light intensity, but not under normal growth conditions (data not shown). Furthermore, shade treatment could lead to regreening of chlorotic leaves (Liu et al., 2017). Thus, it is also interesting to clarify the light intensity thresholds for chlorosis and regreening to understand the mechanisms of tea leaf chlorosis in-depth and for future practical use.

To verify whether *CsClpP3m* is the key causative factor for the chlorotic leaf phenotype in YJX, efforts have been made to complement the chlorotic leaves with transient expression of *CsClpP3 via* leaf infiltration (Sparkes et al., 2006). It is known that exogenous 2% sucrose supply can rescue the albino phenotype in the *Arabidopsis clpp3* mutant (Kim et al., 2013), and nutrition shortage is also involved in the development of chlorotic phenotype (Kim et al., 2013; Liu et al., 2017); thus, our *Agrobacterium* infiltration medium was prepared without the sugar component which was proposed by Sparkes et al. (2006). However, with the sugar-free infiltration medium and the GUS reporter system (GUS: beta-glucuronidase), a slight blue color was observed in the infiltrated tea leaves, which was probably a result of GUS reporter expression (Supplementary Figure 3A). Nevertheless, compared with the leaf blue color in *Nicotiana benthamiana* after infiltration using the same *Agrobacterium* culture (Supplementary Figure 3B), a huge difference in the GUS expression level between tea and *N. benthamiana* was noted, which was probably due to the difference between the two plant species in leaf structure (such as well-developed wax layer in tea leaves) and competence to *Agrobacterium* virulence. Tea leaves contain polyphenol, which is toxic to *Agrobacterium* (Song et al., 2014), and may significantly inhibit the expression of the foreign gene in the infiltrated tea. Therefore, it was not surprising to see the failed attempts to complement the chlorotic leaves in YJX with 35S promoter-driven *CsClpP3*. However, one cannot exclude the possibility that *CsClpP3m* was not the key causative determinant of chlorotic leaves in YJX (Supplementary Figure 3C).

In this study, transcriptional levels of *CsClpP5*, –*6*, and R-ring subunits in chlorotic tea cultivar YJX were all enhanced (*p* < 0.05), except for the non-affected *CsClpP3* and the inhibited *CsClpP4*. Transcriptional quantitative data of different Clp subunits in the RNAi transgenic tobaccos with suppressed *ClpP3* consistently exhibited similar transcriptional alterations, with enhancement in RNAi-4 and –5 lines but not the RNAi-6 line. This probably resulted from the variation among different transgenic lines since it is well known that the different insertion sites and copy numbers of T-DNA may result in significant differences in transgene transcription levels, consequently leading to variant knock-down effects. Moreover, the possible formation of incomplete and malfunctional Clp complex in transgenic tobacco may also result in the alteration of different phenotypes of the RNAi lines. Nevertheless, such distinct transcriptional alterations of Clp genes induced by *ClpP3* suppression in the RNAi transgenic plants suggested that ClpP3 expression could affect the expression of other Clp subunit genes. Previous studies indicated that the expression of Clp genes are tempo-spatially regulated in *Arabidopsis* plants (Olinares et al., 2011b) and in tomato fruits at different developmental stages (D’Andrea et al., 2018). Additionally, it was reported that the transcript levels of P- and R-ring genes are all reduced, rather than enhanced due to null mutation of *CLPR4-1* in *Arabidopsis* (Ramirez Rodriguez et al., 2009), indicating that the mutation of one ClpP gene can possibly affect the transcription of some other ClpP genes. All these findings suggest that transcriptional regulation of the Clp genes could vary with a null mutation of different Clp genes. The transcriptional association between some of the Clp genes might be related to the stoichiometry for the P- and R-rings at the protein level. The ratios for the P- and R-rings are ClpP3:P4:P5:P6 = 1:2:3:1 and ClpP1:R1:R2:R3:R4 = 3:1:1:1:1 (Olinares et al., 2011a). Thus, we speculated that the Clp complex defects induced by malfunctional ClpP3 might alter the transcription of some nuclear Clp genes through plastid-to-nucleus signaling. Further verification studies are required.

Alternative splicing (AS) widely occurs in plants and produces multiple mRNA isoforms from a single gene (Syed et al., 2012); these isoforms frequently contain premature termination codons targeted by nonsense-mediated mRNA decay pathway (Shaul, 2015; Nickless et al., 2017), or generate truncated proteins or polypeptides to negatively regulate the constitutive proteins *via* peptide interference (Yun et al., 2008), which consequently regulates diverse aspects of plant growth and development as demonstrated in maize (Chen et al., 2018). In this study, the intron retention mutation of *CsClpP3m* was found, which produces the malfunctional CsClpP3m and is involved in leaf chlorosis in YJX. The intron retention mutation is the most prevalent form of pre-mRNA AS in plants (Ner-Gaon et al., 2004; Syed et al., 2012); thus, it was not surprising to find this event in tea cultivars tested in this study. Coincident occurrence of the same AS event in several other chlorotic tea cultivars suggests a possible genetic link among these cultivars. The current chlorotic tea genotypes were largely obtained from vegetative tissue mutation and maintained from the propagation of cuttings. The possibility cannot be excluded that the same tea genotype with chlorotic leaves may develop varying chlorotic phenotypes in different geographic locations and thus, are considered as different genotypes. In fact, the genetic backgrounds of most chlorotic tea genotypes and their mother plants are seldom recorded (Wang et al., 2015), unlike some tea cultivars obtained from conventional crosses between known parental cultivars. We have collected the majority of tea genotypes with chlorotic leaves reported so far and expected to reveal the impact of CsClpP3 mutation on chlorotic leaf phenotype.

## Conclusion and Prospects

**FIGURE 7:**
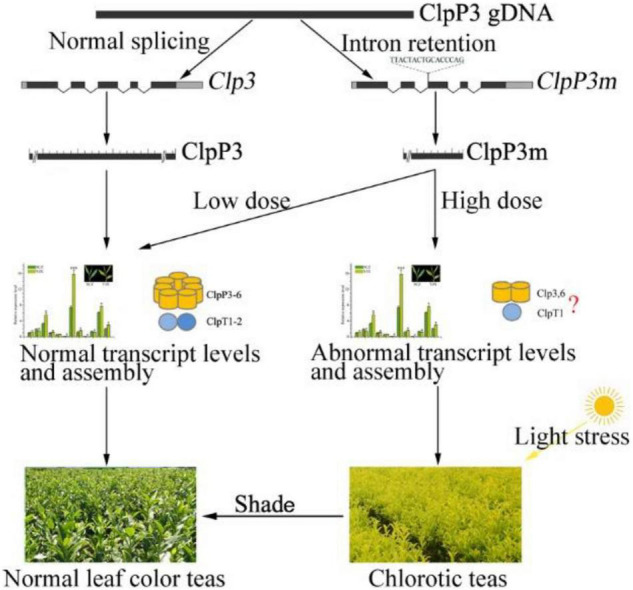
Schematic diagram of the possible mechanism for dose-dependency of ClpP3m in tea leaf chlorosis. The normal Clp complex assembly is adapted from Nishimura and Van Wijk (2015). Abnormal assembly with a red question mark indicates that it was a hypothesis of the authors.

Chlorotic tea cultivars are popular due to the unique flavor of their tea products and show great breeding potential; therefore, uncovering the molecular mechanisms underlying their leaf color phenotype has great theoretical and practical value. In this study, the intron retention variant *CsClpP3m* was shown to be closely involved in YJX leaf chlorosis in a dose-dependent manner, which is likely due to abnormal transcription of Clp subunit genes and CsClpP3m truncation induced Clp complex assembly failure (Figure 7). Moreover, the final formation of leaf chlorosis needs a threshold light intensity causing light stress, and shade treatment can lead to regreening of chlorosis leaves (Figure 7). Further investigations are required to reveal the essential structure of ClpP3 required for Clp complex assembly and the mechanisms underlying the dose-dependent regulation of ClpP3 on the Clp complex function.

## Data Availability Statement

The original contributions presented in the study are included in the article/Supplementary Material, further inquiries can be directed to the corresponding authors.

## Author Contributions

MZ and XL identified ClpP3 intron retention and performed the corresponding molecular analyses including complementation experiments using tea ClpP3 genes. PX performed the experiments on gene cloning and sequence analysis. GL started the project and completed transcriptional analyses of the abnormal Clp component genes and the manuscript draft preparation. SW and GL conceived the project and finalized the manuscript. All authors contributed to the article and approved the submitted version.

## Conflict of Interest

The authors declare that the research was conducted in the absence of any commercial or financial relationships that could be construed as a potential conflict of interest.

## Publisher’s Note

All claims expressed in this article are solely those of the authors and do not necessarily represent those of their affiliated organizations, or those of the publisher, the editors and the reviewers. Any product that may be evaluated in this article, or claim that may be made by its manufacturer, is not guaranteed or endorsed by the publisher.

## Footnotes

1
www.ncbi.nlm.nih.gov

2
http://www.hmmer.org/

3
https://www.ibm.com/cn-zh/analytics/spss-statistics-software

4
tpia.teaplant.org
