# Supplementary material for: The Intron Retention Variant CsClpP3m Is Involved in Leaf Chlorosis in Some Tea Cultivars
Source: Front Plant Sci. 2022 Jan 28;12:804428. doi: 10.3389/fpls.2021.804428 (PMC8831552; doi:10.3389/fpls.2021.804428)
Supplement: Supplementary file 1 [file Data_Sheet_1.pdf]

SUPPLEMENTARY MATERIALS

To the manuscript entitled

The Intron Retention Variant CsClpP3m Is Involved in Leaf Chlorosis in Some Tea Cultivars

Xueyin Luo[1†], Mengxian Zhang[1†], Pei Xu[1], Guofeng Liu[1,2*] and Shu Wei[1*]

[1] State Key Laboratory of Tea Plant Biology and Utilization, Anhui Agricultural University, Hefei, China, [2] Henan Provincial Key Laboratory of Tea Plant Biology, Xinyang Normal University, Xinyang, China

†, Both contributed to this work equally.

*, For correspondence: Shu Wei, weishu@ahau.edu.cn;
Guofeng Liu, liuguof0219@xynu.edu.cn

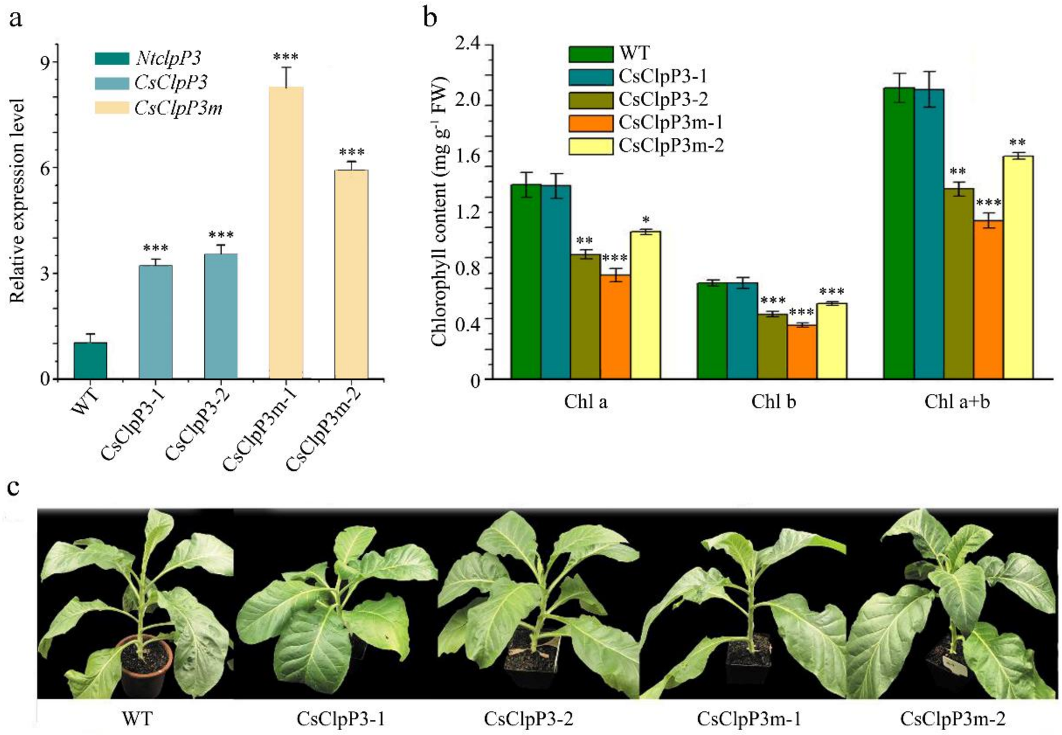

Fig. S1

Fig. S1: Phenotypic analysis of transgenic tobacco over-expressing *CsClpP3* or *CsClpP3m*. a Enhanced transcript levels of *CsClpP3* or *CsClpP3m* in transgenic plants. WT, wild type plants; CsClpP3-1 and -2, transgenic lines expressing *CsClpP3*-YJX; CsClpP3m-1 and -2, transgenic lines expressing *CsClpP3m*-YJX. **b** Chlorophyll contents of transgenic plants over-expressing *CsClpP3* or *CsClpP3m*. **c** No visible difference in leaf color among wild type plant and transgenic tobaccos over-expressing *CsClpP3* or *CsClpP3m*. Statistical analysis was performed using Student *t*-test. *, $p < 0.05$; **, $p < 0.01$; ***, $p < 0.001$.

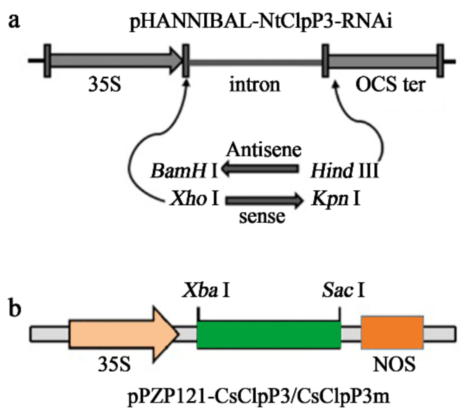

Fig. S2

Fig. S2: Construction of RNAi- NtClpP3 expression cassette using pHANNIBAL intermediate vector (**a**) and pPZP121-CsClpP3/CsClpP3m expression cassettes (**b**).

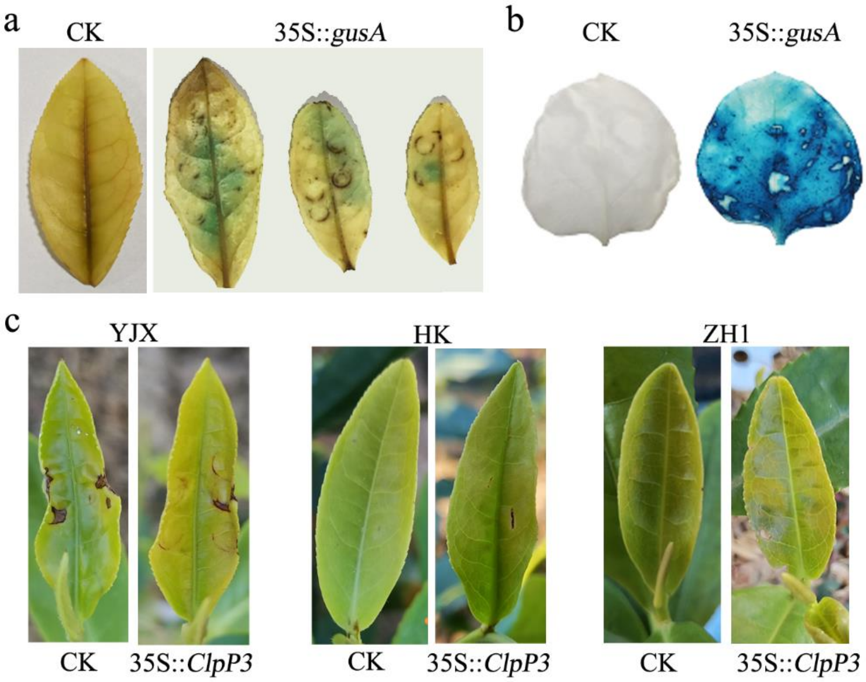

Fig. S3

Fig. S3: Transient expression of pPZP121-CsClpP3 in chlorosis tea leaves. a Transient expression of pPZP121 in chlorotic young tea leaves; **b** Transient expression of pZP121 in tobacco leaves; **c** Leaf infiltration of Agrobacterium GV3101 containing pPZP121-CsClpP3 in YJX; **d** Leaf infiltration of GV3101 containing pPZP121-CsClpP3 in HK; **e** Leaf infiltration of GV3101 containing pPZP121-CsClpP3 in ZH1. CK, control using suspension buffer only for leaf infiltration; 35S::*gusA*, pPZP121; 35S::*ClpP3*, pPZP121-CsClpP3. Transient expression was done according to Sparkes et al. (2006) with minor modifications. In brief, agrobacteria grown to OD_{600} = 0.8-1.0 was used for resuspension with non-sucrose IM medium (AS, Na_3PO_4, MES); the final concentration of resuspended agrobacteria was OD_{600} = 1.0. Leaves were collected 4 d after infiltration. GUS staining was done according to the standard methods.

Table S1: Primers used in the experiments.

Primer name	Primer sequence and usage
	**CsClp gene cloning**
CsclpP3-F	tctagaGAGAGAGAGAGAGAGACAGAGATGGAG
CsclpP3-R	gagctcGTGATAAATCTTACATTGTGTTTGAGG
CsclpP5-F	ggatccATGGCGCATTCGTGCGTGT
CsclpP5-R	ctgcagTTACTGATCTGCACTCGCTGGTAGA
CsclpP6-F	ggatccATGGTAGCTTCAGCCATCTCAGCTC
CsclpP6-R	gtcgacTTAGTATTCTGTTTCCAAGACCCCATCA
CsclpR2-F	ggatccATGGTTATCTCCCTTCATACAACTGGT
CsclpR2-R	gtcgacCTAACCAAGACCTGTTCCTGCATCT
CsclpR4-F	ggatccATGGAGGTCGCCACCATGGC
CsclpR4-R	gtcgacTTAAATAAGTTGTGCTTTTTTCAGGTCAG
CsclpC-F	ggtaccATGGCTGGGGCTTTGGTTCAG
CsclpC-R	ctgcagCTACACAGTGATTGGTTCTGGCAATGA
	**Ntclp cloning**
NtclpP3-sense-F	tttggagaggacacgctcgagCAAGGCTGATGTTTCTACAATCTGC
NtclpP3-sense-R	ttccttaccaattggggtaccTCTTCAGAAGGCAAATTTTTCTTTG
NtclpP3-antisense-F	gaaatcgataagcttggatccTCAGAAGGCAAATTTTTCTTTGC
NtclpP3-antisense-R	tcattaaagcaggactctagaGGCTGATGTTTCTACAATCTGCAT
	**q-RT-PCR in *Camellia sinensis***
c51485-L(ClpP3)	AGCAACAGAGATGAGCATACGAATA
c51485-R(ClpP3)	GTAGGTGCAATTAATCCTGGCTTAC
c48468-L(ClpP4)	CATCAACAGCTTCCATAATCCTTGG
c48468-R(ClpP4)	TCTTGTGACATTGTCCTTGTTATGC
c48000-L(ClpP5)	CTTGCCAAACTCCAGGATAATGATC
c48000-R(ClpP5)	TTGAGAGGATTCATGATAACACCGT
c33064-L(ClpP6)	TCTCTAAGTTGCAGAAATGGTAGCT
c33064-R(ClpP6)	TAGTAGCCTGCTCCTCAAGTTTTAG
c53238-L(ClpR2)	TCAGAATGAGAAGATGGAGACTGTC
c53238-R(ClpR2)	ATATCTATGACAGCTCCACTTGACC
c15808-L(ClpR2-1)	TCTAACTCATGTCTACACTCTGGGA
c15808-R(ClpR2-1)	GTTTACTGTCACAAGTCCTGGATTG
c42794-L(ClpR3)	TGAGGGTTTTGCAATTTATGATGCT
c42794-R(ClpR3)	ATTATTACCTCCTTTGCACGGATGA
c38659-L(ClpR4)	TGTTTACTTGGGCATGTCTCTAGTT
c38659-R(ClpR4)	CTCATAACCTAACTTTTCGCCATCC
c27589-L(ClpC1)	AATAAGATGCCGACACTGGAGGAG
c27589-R(ClpC1)	ATTTGGGTCACACGTTCTATTTGTT
c43453c0-L(ClpC)	AGATCAAAGAAATCGAGCTACAGGT
c43453c0-R(ClpC)	TCGACAATAACTGAATCACCCTCTT
c51361-L-(ClpD)	AAGCATCATCATCATGTTCTTCACC
c51361-R-(ClpD)	TATGAAAGGAGTTGTTGGGTGAGAT
c62560-L-(ClpB1)	GGACCAGAACAAATTGCAAAAGAAG

c62560-R-(ClpB1)	CAACCCTTGATCTCAACATAGCTTC
c41573-L-(ClpB3)	TTCCAGTATTATTCCTCACGCTCTT
c41573-R-(ClpB3)	TCATTTGTAGCTTGCAGAAGTGATG
	**Quantitative RT-PCR for *Nicotiana tabacum*[a]**
NtClpP3_F-qPCR	TCTCGGAACTGGGATGTTTC
NtClpP3_R-qPCR	GATTCCCATCCCAGCAGTTA
NtClpP4_F-qPCR	CCTCACTGTTCCTCCCTCAA
NtClpP4_R-Qpcr	GAGAAGGGGGTTTTTGAAGC
NtClpP5_F-qPCR	TGTTGATCCCACAAAGGACA
NtClpP5_R-qPCR	CCAAGAGGCTGGTGAATCAT
NtClpP6_F-qPCR	GAGAACCCCGTTAAAGCTCA
NtClpP6_R-qPCR	AGTTGCGAGGGTCACAAGTT
NtClpR1_F-qPCR	ATAACCCAGTACGGCGACAG
NtClpR1_R-qPCR	GGCATGCCCAGATAGACAAT
NtClpR2_F-qPCR	GGGATTGCAGCGTCTAATGT
NtClpR2_R-qPCR	CACGTTCACGGTAAAGAGCA
NtClpR3_F-qPCR	GTGCCAGCAGTCACAGAGTT
NtClpR3_R-qPCR	CAGCTGCAAGCAAGAGACAC
NtClpR4_F-qPCR	CTCTTCGCCCTTCTTCCTCT
NtClpR4_R-qPCR	TGGCTTTTCCTCATCCTCAT
NtClpS_F-qPCR	TCCCCATCAAATCTTCCAAC
NtClpS_R-qPCR	GAATTCAGATTCACGCCCAG
NtClpT1_F-qPCR	TCAACTTCGACTTCTTACAA
NtClpT1_R-qPCR	GCCGACCATTTGGGCTGTAT
NtClpT2_F-qPCR	TAGGACCCAATTCGTCGTTC
NtClpT2_R-qPCR	ATTCACCCATGGCAAATGAT
NtClpC_F-qPCR	GGCGTCGACCTTACACTGTT
NtClpC_R-qPCR	TCACACTGCTTCCGACATTC
NtClpD_F-qPCR	AATGCTGCTGTGCAACTGTC
NtClpD_R-qPCR	TGCTTGCCAAGATCACTTCA
	**Cloning in *Arabidopsis mutant***
AtclpP3-F	TCCCAATCCGAAACCATAGAATCC
AtclpP3-R	GGTACAAACACAGGAAATGCAACGT
pROK2-LBb1	GCGTGGACCGCTTGCTGCA

[a] Primers used for tobacco were taken from Moreno et al. *J. Exp. Bot.* **68**. 2199–2218(2017).
